# Effect of nanoparticle based intra-canal medicaments on root dentin micro-hardness

**DOI:** 10.6026/97320630018226

**Published:** 2022-03-31

**Authors:** Iffat Nasim, Krishna Kanth Jaju, M Shamly, V Vishnupriya, Zohra Jabin

**Affiliations:** 1 Department of Conservative Dentistry and Endodontics, Saveetha Dental College and Hospitals, Saveetha Institute of Medical and Technical Sciences, Saveetha University, India; 2 Department of Biochemistry, Saveetha Dental College and Hospitals, Saveetha Institute of Medical and Technical Sciences, Saveetha University, Chennai - 600 077, India; 3 Divya Jyoti College of Dental Sciences, Modinagar, Uttar Pradesh, India

**Keywords:** nanoparticle, intra-canal, medicaments, root dentin, micro-hardness

## Abstract

Complete eradication of bacterial infection from the root canal with minimum or no effect on the microhardness of the dentin is desirable for the success of endodontic treatment. The effect of nanoparticle based intracanal medicaments on the root dentine microhardness was assessed. The medicaments used were combination of calcium hydroxide - silver nanoparticles, Combination of graphene oxide - silver nanoparticles, Calcium hydroxide and a control group. Vickers hardness value were evaluated and compared at coronal, middle and apical third level. Graphene oxide - silver nanoparticles based intracanal medicament showed least effect on the microhardness of the root dentin compared to calcium hydroxide and calcium hydroxide combined with silver nanoparticle based intracanal medicaments.

## Background:

The main objectives of endodontic therapy are root canal disinfection and elimination of bacteria present in periapical region [1]. The complexity of root canal anatomy prevents the instrumentation into the intricacies of the root canal system which may attribute to failure of endodontic treatment [2]. Shaping of root canals by hand and rotary files cannot completely eliminate bacteria from the intricate areas such as the lateral canals and isthmus [3]. That's why use of intra canal medicaments and effective irrigation is essential which may aid in eradication of bacteria and facilitate healing of periapical lesions [4]. Calcium hydroxide Ca (OH) 2 has a long track record in history and is the most commonly used intracanal medicament during endodontic therapy. In the treatment of chronic periapical lesions, it has shown promising results. Ca (OH)2 is effective in neutralizing bacterial endotoxins and promoting periapical healing. The organic support of the dentin matrix is decreased after exposure to calcium hydroxide, which may be attributed to its high pH. Furthermore, high alkalinity may lead to disintegration of protein structure, which may affect mechanical characteristics of dentin. Dentin microhardness is reduces due to breakdown of linkage between the collagen fibres and hydroxyapatite crystals [5]. Silver nanoparticles have been widely used in dentistry and biomedicine, due to its antibacterial properties. It has been tested for application as retrograde filling material, restorative material, caries inhibitory solution etc. Silver exerts its antibacterial effect by interacting with sulfhydryl groups of proteins and DNA, altering the hydrogen bonding/respiratory chain, unwinding DNA, and interfering with cell-wall synthesis/cell division of bacteria [6]. Silver nanoparticles (Ag-NPs) disrupt the bacterial membrane and increase permeability thereby resulting in expulsion of cell constituents. The combination of silver nanoparticles with calcium hydroxide has shown promising results in eradication of microorganisms from the root canal system [7]. Graphene, a carbon allotrope, is the thinnest material which can form a crystal lattice with no structural dislocations. The antibacterial property of silver nanoparticles was enhanced by adding graphene, and there were little cytotoxic effects on bone and soft tissues. Graphene oxide nanoparticles are extremely effective in elimination of Streptococcus mutans [8]. Antibacterial compounds derived from various natural and synthetic sources have been thoroughly investigated and are proposed as intracanal drugs. However, adequate property determination of these drugs is required prior to their usage. Microhardness testing can be used to infer indications of mineral loss or gain in dental hard tissues [9]. Calcium hydroxide based intracanal medicaments affects root dentin, its physical characteristics and subsequently compromises the fracture resistance of the tooth [10]. However, there is no sufficient information about the effect of these novel intracanal drugs on fracture resistance of root dentin. The present study aims to evaluate the effect these intracanal medicaments on microhardness of root dentin.

## Material & Methods:

### Sample Preparation:

The study protocol was approved by the institutional ethical committee. Sixty recently extracted single-rooted human premolar teeth with straight roots, closed apices, no caries, cracks, fractures, restorations or resorption were included in this study. Radiograph was taken to confirm the presence of a single canal. Calculus and stains were removed and the teeth were stored in phosphate buffered saline until used. Teeth were decoronated at cemento-enamel junction (CEJ) using diamond disc (Kerr Dental, Europe) at low speed under water coolant.15 K-file (Dentsply, Maillefer, Ballaigues, Switzerland) was introduced into the canal and working length was determined 0.5 mm short of the apex. Cleaning and shaping was done according to the manufacturer's instructions using ProTaper Gold rotary files and Dentsply X-smart plus endomotor (Dentsply Maillefer, Ballaigues, Switzerland) (S1-F3). RC-Prep (Premier Dental Products, USA) was used as lubricant and 5 - 10 mL of 2.5% sodium hypochlorite (NaOCl) was used as an irrigant. A final rinse was with 5.25% NaOCl and 3mL of 17% ethylenediaminetetraacetic acid (EDTA) was carried out for five minutes, Irritant activation was done using Endoactivator (DentsplyMaillefer, Ballaigues, Switzerland). Each canal was then rinsed with 5 mL saline and dried using paper points. The specimens were sectioned longitudinally in a bucco-lingual direction using a diamond disk at low speed, passing through the canal after cleaning and shaping. The root segments were mounted horizontally using auto polymerizing acrylic resin leaving the canal exposed. The dentin surface of the specimens was polished with carbide abrasive papers under distilled water to remove surface scratches to achieve smooth glossy surface.

Baseline microhardness values were recorded for each sample before application of the respective intracanal medicaments. The samples were then randomly divided to 4 treatments groups (n=15).

Group 1 - Calcium hydroxide - silver nanoparticles

Group 2 - Graphene oxide - silver nanoparticles

Group 3 - Calcium hydroxide

Group 4 - Saline

### Preparation of intracanal medicaments:

Silver Nanoparticles preparation:

To silver nitrate solution, the plant extract of Andrographis paniculata and Ocimum sanctum Linn was added which served as reducing and stabilizing agent, the prepared solution was centrifuged, filtered and biosynthesized silver nanoparticles were obtained.

For Ca (OH) 2 - AgNP group, Ca (OH) 2 powder (PREVEST DENPRO LTD, INDIA) was mixed with biosynthesized AgNP suspension in 1:1 ratio.

For Graphene oxide - silver nanoparticle group, 0.5g of graphite nano powder (Sisco Research Laboratories, Maharashtra, and India.) and 0.1 g of sodium hydroxide (MERCK, Mumbai, India.) was mixed with biosynthesized AgNP suspension in 1:1 ratio.

For Ca (OH) 2 group, Ca (OH) 2 powder (PREVEST DENPRO LTD, INDIA) was mixed with distilled water.

The respective medicaments in each group were applied to the root canals. The specimen was then kept in the incubator at 37°C and 100% humidity for 7 days.

### Evaluation of dentin microhardness:

Baseline microhardness value was measured at magnification of 100X using Vickers Microhardness Tester (Model LM-100, FM 1159 LECO Corporation Michigan, and USA) 25 g load of load was for applied for 10 s. The microhardness values were determined at coronal, middle and apical third. Three measurements were recorded for each section. Intracanal medicaments were then applied to each specimen according to the assigned group. The specimen was stored for 1 week in an incubator at 37°C. After 1-week intracanal medicaments were removed by rinsing with saline, then microhardness was tested again with the same parameters for each specimen and compared with the baseline values.

### Statistical Analysis:

Data was analyzed using SPSS software version 21. Paired wise sample t test was used for inter group comparison. ANOVA test and Post Hoc Tukey was used for intra group comparison. P value of < 0.05 was considered significant.

## Results and Discussion:

All the groups except graphene oxide - silver nano particle group showed reduction in mean microhardness with a statistically significant difference (p < 0.05). There was a significant difference among the groups at coronal third, middle third and apical third (P<0.05). In between groups the coronal third and middle third region graphene oxide - silver nanoparticles showed the highest mean microhardness values, in the apical third region control group showed the highest mean microhardness values. Table 1(see PDF) Major goal of endodontic therapy is complete bacterial eradication from the root canal system. In teeth with apical periodontitis after chemo-mechanical preparation in the form of cleaning and shaping, use of intracanal medicaments is mandatory to cut off the source of nutrients for bacteria, prevent their growth, and stop their ingress into the root canal [11]. Till date Calcium hydroxide is the most commonly used intracanal medicament for root canal disinfection. The calcium hydroxide exerts its antimicrobial activity by the release of hydroxyl ions. Hydroxyl ions are highly reactive, oxidant free radicals that interact with a wide variety of biomolecules. Ca (OH) 2 acts on bacterial cells by damaging the cytoplasmic membrane, protein denaturation and damage to the DNA [12]. However, due to the inability of Ca (OH) 2 to have antimicrobial activity against all pathogens, limitations in its physicochemical properties, and a comparatively greater penetrability of the microorganisms into the dentin, research has been conducted on alternative materials such as chlorhexidine, metronidazole, bioactive glass, nanoparticles etc. to be proposed as intracanal medicaments. Nanoparticles have shown promising properties in the field of medicine as well in development of newer pharmaceutical products. As a catalyst, sensors, and adsorbents they have shown significant activity. The diameter of these nanoparticles is <1 mm. Their small size gives them the advantage of having a larger surface area and higher reactivity [13]. Nanoparticles have been tested in various forms within the root canal and have shown promising results. Antimicrobial activity of nanoparticles against common endodontic pathogens has shown superior results. Silver nanoparticles (AgNPs) possess antimicrobial action against bacteria and viruses. They possess bactericidal action against Gram-positive and Gram-negative bacteria. AgNPs penetrate into the bacterial cell wall and increase the cell wall permeability, thus destroying the integrity of the bacterial membrane. AgNPs have been proven to be effective against oral pathogens; they have better antibacterial action against Streptococcus mutans when compared with CHX. AgNPs require a longer period of interaction with the microorganisms within the biological tissues to exert a robust antimicrobial action. Hence, AgNPs have been formulated as gel, for a potential mode of application as an intracanal medicament. The AgNP gel proved to be more effective than Ca (OH) 2 against *E. faecalis* biofilm after a period of 7 days, substantiating their effectiveness in the gel form for potential use as an intracanal medicament in eliminating *E. faecalis* . AgNPs have also been tested in combination with Ca (OH) 2 against *E. faecalis* . AgNP/Ca (OH) 2 combination proved to be more effective than Ca (OH) 2 alone, after 1 and 7 days as an intracanal medicament in eliminating *E. faecalis* [14]. Graphene oxide contains a hexagonal carbon structure containing oxygen-based functional groups such as hydroxyl (OH), alkoxy (C-O-C), carbonyl (C-O), carboxylic acid (COOH), and others. Graphene oxide exhibits hydrophilic property that makes it a water-soluble nanomaterial due to the presence of these functional groups. Graphene oxide has high conductivity and shows diverse applications in the field such as sensors, anticancer properties, electronics, biomedicine antimicrobial coatings, photocatalytic activity, water decontamination, solar desalination, and drug delivery [15]. Silver (Ag) is the most conductive and reactive of the transition elements, and it has recently been utilized to fabricate silver-doped graphene oxide with desirable characteristics such as low resistance, excellent dispersion, and increased mechanical strength. AgNPs have recently received a lot of attention in antibacterial applications due to their bactericidal properties. The antibacterial effectiveness improves when graphene is incorporated into silver nanoparticles [16]. Dentin microhardness determines flexural strength and tensile strength of the tooth. The use of intracanal medicaments and instrumentation causes changes in the structure of the root dentin leading to a weak tooth structure. The assessment of dentin microhardness is mandatory to determine the effectiveness of any new intracanal medicament.

In present study, microhardness measurements were recorded for the root canal dentin at coronal, middle and apical third level. A Mean Vickers hardness number (VHN) was calculated for each specimen. Vickers microhardness test was selected for this study as it permits to evaluate surface changes of deeper dental hard tissues [17]. Other hardness tests such as Knoop hardness are only restricted to evaluate the microhardness of only the superficial dentin at 0.1 mm and not advocated for deep dentin. In the present study, a combination of graphene oxide - silver nanoparticles based intracanal medicament exhibited less reduction in microhardness as compared to other groups. The increase in dentine microhardness found in the samples treated with the graphene combined silver nanoparticles group could be attributed to the deposition of silver nanoparticles on the dentine surface and inside the dentinal tubules [18]. Aqueous dispersions of graphene oxide were found to be stable in the pH range of 4-12, with excellent dispersibility in the range of 7-11 [19]. This may have been attributed for better microhardness property in the graphene oxide combined silver nanoparticle medicament group. Although Ca (OH) 2 is most frequently utilized as an intracanal medicament and is considered as the gold standard, reduction in root dentin microhardness at the moderate to intermediate level has been reported [20]. The possible explanation for this destruction could be denaturing of the organic matrix or breakdown of the dentin inorganic structure due to the highly alkaline inorganic molecule with pH of 11.8 [21].

## Conclusion:

Combination of Graphene oxide - silver nanoparticles showed least effects on microhardness of the root dentin compared to calcium hydroxide and calcium hydroxide - silver nanoparticle based intracanal medicament.
